# Supplementary material for: Microsatellite analysis reveals connectivity among geographically distant transmission zones of Plasmodium vivax in the Peruvian Amazon: A critical barrier to regional malaria elimination
Source: PLoS Negl Trop Dis. 2019 Nov 11;13(11):e0007876. doi: 10.1371/journal.pntd.0007876 (PMC6874088; doi:10.1371/journal.pntd.0007876)
Supplement: S1 Checklist — (DOC) [file pntd.0007876.s001.doc]

STROBE Statement—checklist of items that should be included in reports of observational studies

|  | Item No | Recommendation |
| --- | --- | --- |
| **Title and abstract** | 1 | (*a*) The study’s design with a commonly used term in the title or the abstract: Abstract section, paragraph 1. |
|  |  | (*b*) An informative and balanced summary of what was done and what was found: Abstract and Author summary sections. |
| Introduction | | |
| Background/rationale | 2 | Explain the scientific background and rationale for the investigation being reported: Introduction section, paragraphs 1 – 4. |
| Objectives | 3 | State specific objectives, including any prespecified hypotheses: Introduction section, paragraph 4. |
| Methods | | |
| Study design | 4 | Present key elements of study design early in the paper: Methods section, paragraph 3. |
| Setting | 5 | Describe the setting, locations, and relevant dates, including periods of recruitment, exposure, follow-up, and data collection: Methods section, paragraphs 2 and 3. |
| Participants | 6 | (*a*) The eligibility criteria, and the sources and methods of selection of participants. Describe methods of follow-up: Methods section, paragraph 3. |
| Variables | 7 | Outcomes:  *Plasmodium vivax* positive infections in Methods section, paragraphs 4 and 5.  Parasite density in Methods section, paragraph 5.  Genotypic information in Methods section, paragraphs 6, 7 and 8.  Genetic metrics in Methods section, paragraphs 9, 10 and 11.  Haplotypic diversity (Simpson index) in Methods section, paragraph 9.  Heterozygosity in Methods section, paragraph 9.  Population mutation rate in Methods section, paragraph 9.  Linkage disequilibrium in Methods section, paragraph 9.  Pairwise population differentiation in Methods section, paragraph 10.  Source of population differentiation (AMOVA) in Methods section, paragraph 10.  Estimation of the number of parasite genetic populations in Methods section, paragraph 11.  Ancestry coefficient per each sample in Methods section, paragraph 11.  Genetic relatedness of genotypes (Minimum spanning network) in Methods section, paragraph 11.  Diagnostic criteria:  *Plasmodium vivax* diagnosis in Methods section, paragraph 4 and 5.  Sample assignation to specific parasite genetic populations in Methods section, paragraph 11.  Homologous and heterologous recurrent episodes in Methods section, paragraph 12. |
| Data sources/ measurement | 8* | Comparability of assessment methods in Methods section, paragraph 9. |
| Bias | 9 | Efforts to address potential sources of bias Methods section, paragraph 5, 8 and 9. |
| Study size | 10 | Study size in Methods section, paragraph 2. |
| Quantitative variables | 11 | How quantitative variables were handled in the analyses. If applicable, describe which groupings were chosen and why.  Determination of the parasite density in Methods section, paragraph 5.  Differences in parasite density among study areas and between homologous and heterologous infections in Methods section, paragraph 12.  Comparability of assessment methods and differences in Heterozygosity among study areas and among different seasons in Methods section, paragraph 9. |
| Statistical methods | 12 | (*a*) All statistical and population genetics methods are described in Methods section, paragraph 9, 10, 11 and 12. |
|  |  | (*b*) Methods used to examine subpopulation structure are described in Methods section, paragraph 11. |
|  |  | (*c*) Missing alleles are described in Methods section, paragraph 8. |

Continued on next page

| Results | | |
| --- | --- | --- |
| Participants | 13* | (a) The number of genotyped samples is given in Results section, paragraph 1.  The number of individuals with recurrent episodes is described Results section, paragraph 11. |
|  |  | (b) Non-selection at each stage is Results section, paragraph 1. |
|  |  | (c) Flow diagram in Results section, paragraph 1. |
| Descriptive data | 14* | (a) Genetic diversity description at spatial scale is in Results section, paragraphs 2, 3 and 4.  Population structure results are described in Results section, paragraph 6 - 10. |
|  |  | (b) Participants with missing alleles and missing epidemiological data are in S3 and S4 tables. |
|  |  | (c) The temporal change of genetic diversity is described in Results section, paragraph 5. Temporal change of the parasite population structure is described in Results section, paragraphs 8, 9 and 10. Dynamic of parasite clone replacement is described in Results section, paragraphs 11, 12 and 13. |
| Outcome data | 15* | Statistical differences in the temporal change of genetic diversity is described in Results section, paragraph 5. Temporal change of the parasite population structure and change in parasite genetic diversity by subpopulations over time in Results section, paragraphs 8, 9 and 10. Dynamic of parasite clone replacement and differences in parasite load in individuals with recurrent infections in Results section, paragraphs 11, 12 and 13. |
| Main results | 16 | (*a*) Estimates of genetic diversity by geographic location, by season and by genetic populations are given in Results section, paragraphs 2 – 5 and 8 – 10. |
| Other analyses | 17 | Dynamic of parasite clone replacement is described in Results section, paragraphs 11, 12 and 13. |
| Discussion | | |
| Key results | 18 | The summary of key results is presented in Discussion section, paragraph 1. |
| Limitations | 19 | The limitations of the study are presented in Discussion section, paragraph 5. |
| Interpretation | 20 | A cautious overall interpretation of results is presented in Discussion section, paragraphs 1-4. |
| Generalisability | 21 | The generalisability (external validity) of the study results is discussed in Discussion section, paragraph 6. |
| Other information | | |
| Funding | 22 | This study was funded by National Institutes of Health-National Institute of Allergy and  Infectious Diseases (NIH-NIAID) U19AI089681 to JMV (https://www.niaid.nih.gov); and  Training Grant 5D43TW007120 (https://www.fic.nih.gov). The funders had no  role in study design, data collection and analysis, decision to publish, or preparation of  the manuscript. |

*Give information separately for cases and controls in case-control studies and, if applicable, for exposed and unexposed groups in cohort and cross-sectional studies.

**Note:** An Explanation and Elaboration article discusses each checklist item and gives methodological background and published examples of transparent reporting. The STROBE checklist is best used in conjunction with this article (freely available on the Web sites of PLoS Medicine at http://www.plosmedicine.org/, Annals of Internal Medicine at http://www.annals.org/, and Epidemiology at http://www.epidem.com/). Information on the STROBE Initiative is available at www.strobe-statement.org.
