# Supplementary figures and images for: Microsatellite analysis reveals connectivity among geographically distant transmission zones of Plasmodium vivax in the Peruvian Amazon: A critical barrier to regional malaria elimination
Source: PLoS Negl Trop Dis. 2019 Nov 11;13(11):e0007876. doi: 10.1371/journal.pntd.0007876 (PMC6874088; doi:10.1371/journal.pntd.0007876)

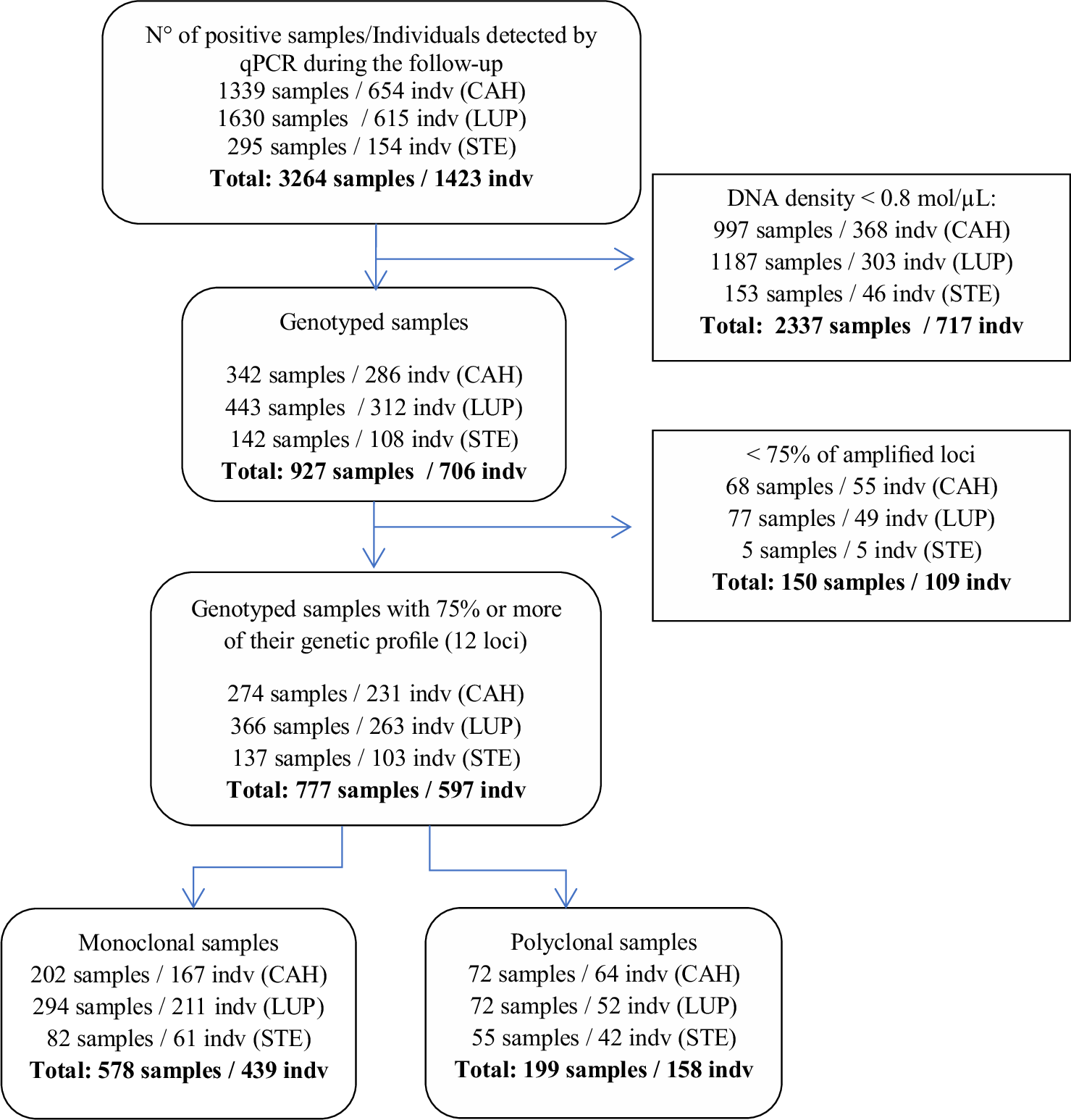

Supplement: S1 Fig — Samples with DNA concentration below 0.8 mol/μL were discarded because they were under PCR limit of detection. Samples with less than 75% of their genetic profile (less than 12 loci amplified) were also discarded. Selected samples were classified as monoclonal or polyclonal according to the number of alleles found per each locus. (TIF) [file pntd.0007876.s002.tif]

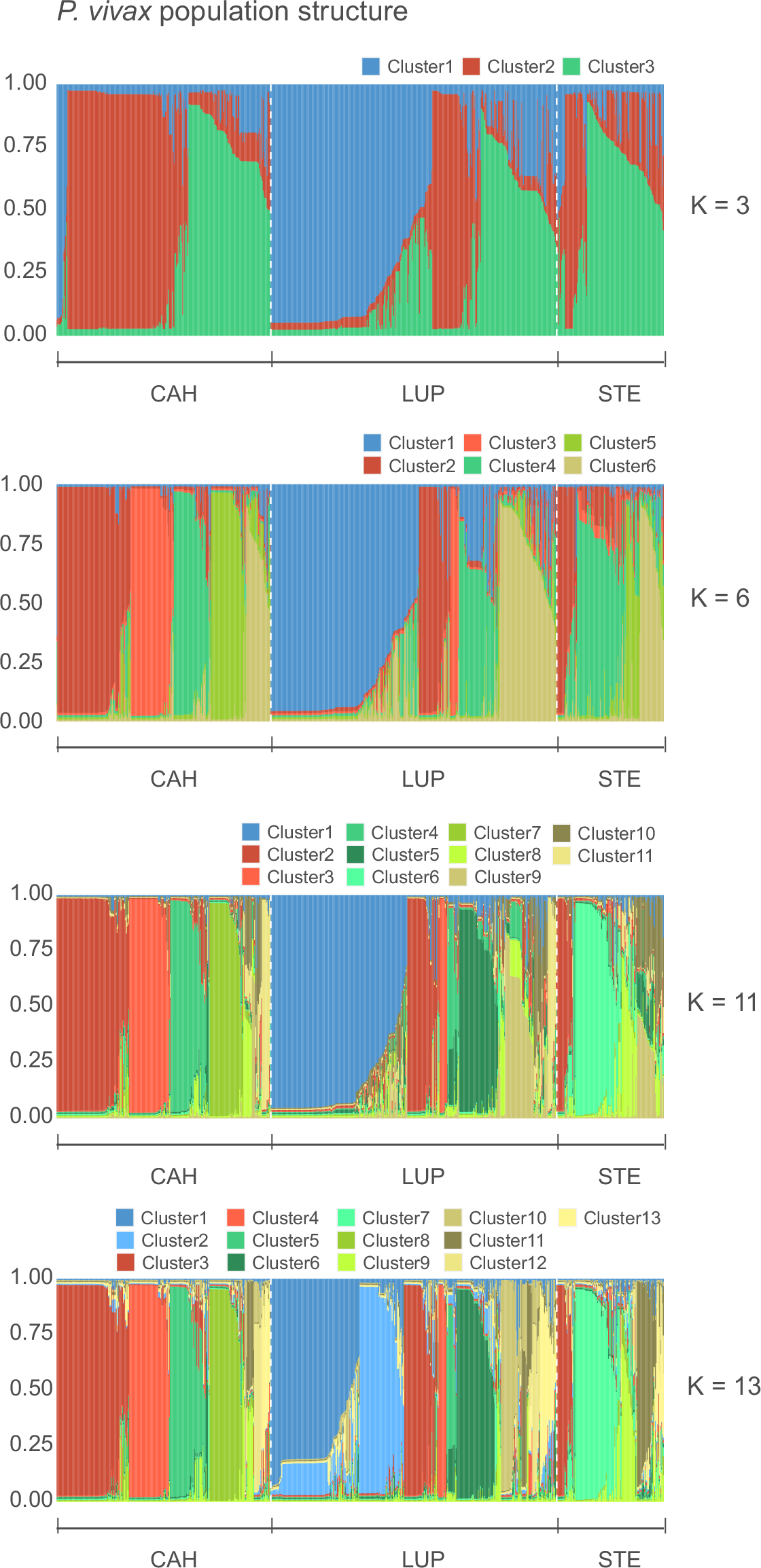

Supplement: S2 Fig — Each color represents an ancestral population, and samples have the same order in each graph. (TIF) [file pntd.0007876.s003.tif]

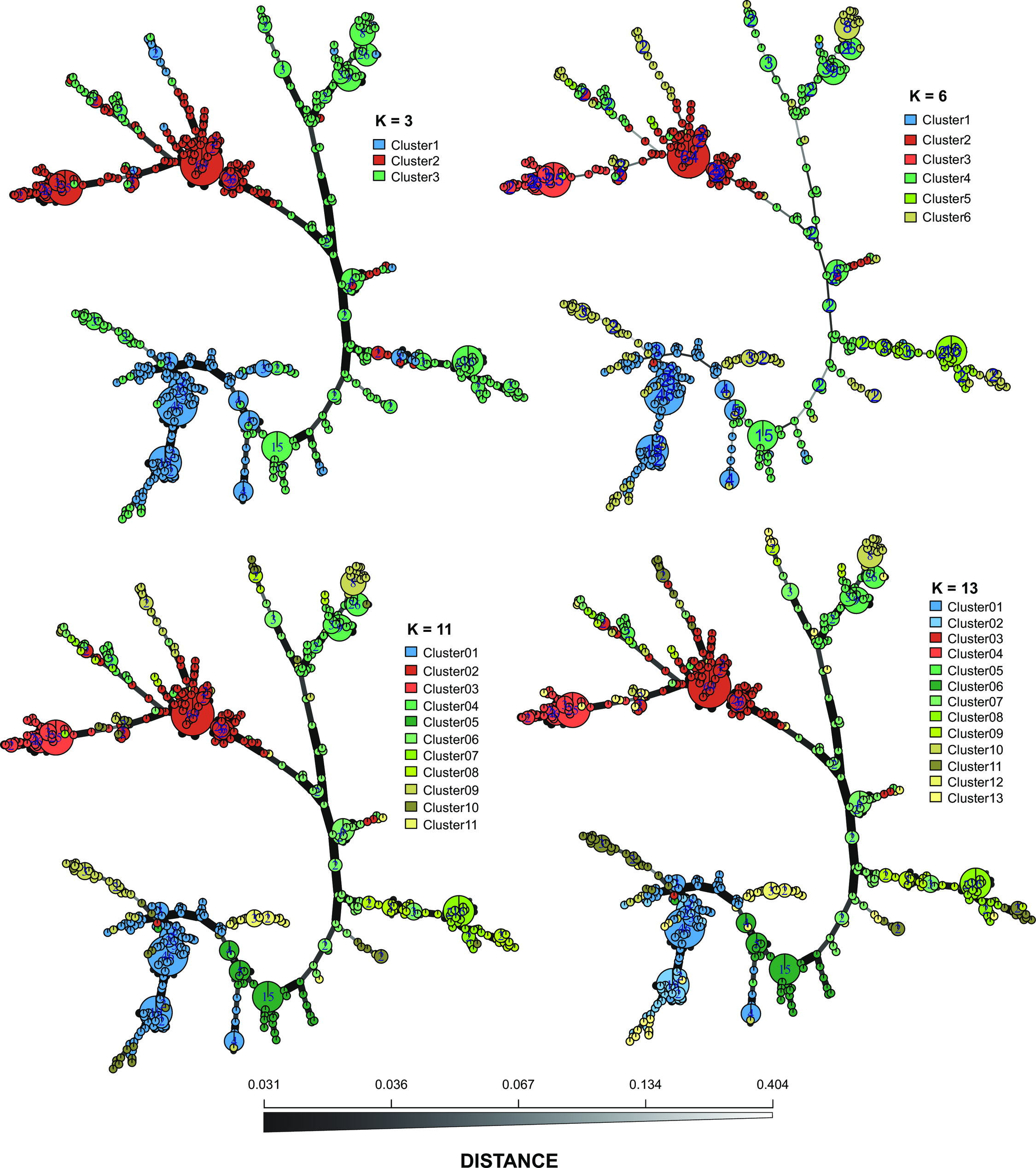

Supplement: S3 Fig — Color denotes parasite clusters according to the population structure analysis for each K. The circle size corresponds to the number of total individuals with the same haplotype. Branch darkness is proportional to inferred genetic distance between haplotypes. (TIF) [file pntd.0007876.s004.tif]
