# Supplementary material for: Microsatellite analysis reveals connectivity among geographically distant transmission zones of Plasmodium vivax in the Peruvian Amazon: A critical barrier to regional malaria elimination
Source: PLoS Negl Trop Dis. 2019 Nov 11;13(11):e0007876. doi: 10.1371/journal.pntd.0007876 (PMC6874088; doi:10.1371/journal.pntd.0007876)
Supplement: Attachment — Submitted filename: Response_to_reviewers.pdf [file pntd.0007876.s012.pdf]

UNIVERSIDAD PERUANA
CAYETANO HEREDIA

October 11, 2019

Dear reviewers and editors,

We deeply appreciate the comments and suggestions made by you to improve the quality of our manuscript titled "Microsatellite analysis reveals connectivity among geographically distant transmission zones of *Plasmodium vivax* in the Peruvian Amazon: A critical barrier to regional malaria elimination".

We believe that the suggestions made will help to better understand the decisions made in the experimental design, especially in the definition of alleles, polyclonal infections and recurrent infections. We also consider that the new modifications more adequately define the scope of some of our results, as is the case of recurrent infections, which due to the strong population structure and clonal expansion of haplotypes at local level, makes it impossible to identify whether the cause of these infections is due to a new mosquito bite, a relapse or a recrudescence. Finally, a bottleneck analysis was added which confirmed the lack of effectiveness of the interventions carried out on the study areas.

Below are the 13 suggestions made by the reviewers and the changes made:

1. **Reviewer #1 Lines 116-130:** The authors present population movements whose intensity seems to be known. Are authors could give references and quantification if possible, of these population movements?

   Unfortunately, the information was obtained by direct communication with the inhabitants of the areas. We have also obtained information from the census which has been already cited, however, only information from the month previous from the census was collected which no reflects the real pattern of human movement. For these reasons, quantitative data is not available at this moment.

2. **Reviewer #1 Lines 206-209:** Can the authors explicit how they define their threshold of 50RFUs for the allele definition. Have they realized any replications of the experiment for a sample set (in triplicates for example)?

   Yes, we perform replicates for 4 control samples in all analyzed plates, this information has been added in methods (see lines 208-214) and in the results (lines 300 -320).

3. **Reviewer #1 Lines 211-212:** The authors explicit that only genotyped samples with 75% or more of their genetic profile are considered. Do they also define a threshold of missing data per loci or all 12 selected loci were analyzed whatever their profile and succeed rate of amplification?

   Yes, we use a threshold of 10%, and all loci meet this requirement.

4. **Reviewer #1 Lines 259-261**: Could the author explain deeply their classification of recurrent infections? Does it was only based on the follow-up assuming that all new cases in a patient is a recurrent infection?

   Yes, all new infections detected by qPCR in a patient were classified as a recurrent infection regardless of the origin or cause of the recurrence (new infection, relapse, the persistence of

the primary infection). This information has been added in lines 286 - 288. We know that this case definition is limited and does not specify the cause of the recurrent infection. However, our intention is not to differentiate between reinfections, relapses or other causes, but to explore how parasite density evolves in people where the same parasite clone persists (homologous recurrences) or in people who have a replacement of the clone that caused the initial infection (heterologous recurrences).

5. **Reviewer #1 Lines 422-440:** Was it possible (considering epidemiological data) to rule out the probability that a homologous infection was caused by a new infection rather than by a relapse? Likewise, studies show that the genetic profile of relapse may differ from that of the first infection, did the authors observe this?

   Information about how many recurrent infections were homologous or heterologous is presented in the previous paragraph (lines 470 - 484). Unfortunately, we have not developed yet a statistical framework to differentiate between new infections and relapses using the epidemiological and genotyping data.

6. **Reviewer #1 Lines 480-482:** Given the resilient nature of P. vivax parasites, did the authors also observed a specific genetic signature (bottleneck per se) supporting the limited efficacy of the interventions?

   Yes, that is correct. For that reason, we have included this analysis in methods (lines 277 - 285) and results (lines 451 - 466). Three new tables have been added (Table 3, S10 and S11 tables). In summary, we didn't find any signal of bottleneck in the population, instead of that our data reveals a recent population size expansion (a deficit of heterozygosity).

7. **Reviewer #3 Line 58:** it is not clear what "introduction" is referred to.

   The word "introduction" refers to the entry of a new parasite clone or subpopulation into the geographic area of LUP, this information has been added to the manuscript in order to avoid confusion (lines 58 and 59).

8. **Reviewer #3:** It is not clear what is the exact distance between the study locations and how they relate mobility wise. This should be addressed in the Methods section.

   In order to clarify the distance between study areas, the travel duration (in minutes or hours) and the means of transport used from Iquitos to each study area has been added in the section of "Study area and population".

9. **Reviewer #3 Lines 140-141:** it should be: from March 1st, 2013 to February 28th, 2014.

   Yes, it was a typing error and the correction has already been made (line 144).

10. **Reviewer #3 Line 207-208:** The authors consider an allele any secondary peak higher that one fifth of the main peak. Why did they choose this measure, taking into account that other studies use more than one third. An explanation or reference is needed.

UNIVERSIDAD PERUANA
CAYETANO HEREDIA

The explanation and reference have been added in lines 215 - 230. Basically, the intention of reducing the threshold was to increase the sensitivity for the detection of polyclonal infections, as most secondary clones could have been in a proportion of less than one third. In order to avoid confusion with artifacts, stutters or cross-contamination three measures were taken: First, the estimated allele size of the secondary peak should have coincided with alleles detected on monoclonal samples; second, 4 control samples (including one polyclonal mock sample) were analyzed 12 times by the 16 markers in order to quantify the amount of cross-contamination and identify and characterize the stutters of each marker; third, the height of the secondary peak should have been greater than the expected stutter peak.

11. **Reviewer #3 Lines 315-329:** Figure 2 is not correctly cited. The order of the parts of the figure is incorrect. Please re check the order of the figure and change in the text or change the figure itself.

    We recheck the order of figure 2 and it seems ok, but in order to make it clearer, we have changed the order of the information in the text (lines 365 - 379) and modified the figure.

12. **Reviewer #3 Lines 466-467:** The authors talk about 3 genetic subpopulations (assuming K=3). Is this correct? Shouldn't it be K=6?

    Yes, that is correct. We have changed the information in lines 533 and 534.

13. **Reviewer #3 Line 537:** should be emphasizes.
    That is correct, we have already change it in line 604.

We hope you agree with the changes made to the manuscript, and we hope to hear good news from you soon.

Sincerely yours,

Paulo Manrique Valverde, Mcs.
Research Assistant Amazonian ICEMR
Departamento de Ciencias Celulares y Moleculares
Universidad Peruana Cayetano Heredia
Av. Honorio Delgado 430, SMP, Lima, PERU
Phone: +51-1-3190000, ext. 233317
paulo.manrique.v@upch.pe/paulonvnv@gmail.com
